# Skin and Genito-Urinary Diseases

**Published:** 1893-06

**Authors:** 


### SKIN AND GENITO-URINARY DISEASES.

EDITED BY DR. M. B. HUTCHINS.

### SEBACEOUS GLANDS AND SWEAT GLANDS.

On November 24, 1892, Dr. Wallace Beatty, in his inaugural address, upon becoming President of the Dublin Biological Association, discussed at length the function of the sweat and sebaceous glands (*British Journal of Dermatology, April*, 1893). Unna had adopted the view and arguments brought out in 1856 by Meissner, that the sebaceous glands (those in the mouths of which we see "black heads") secreted a substance for the oiling of the hairs. The coil of the sweat gland, according to Unna, secreted fat, the duct of the gland received the watery part of the sweat from the surrounding tissues. The oil, or fat, lubricates the skin and the watery part results from the necessity of cutaneous transpiration.

Dr. Beatty had made numerous experiments upon man and animals and had examined many sections of skin from all for the purpose of confirming or refuting Dr. Unna's views. These experiments and examinations were thorough and complete and the speaker announced that they did not sustain the views of Dr. Unna. So we have the matter practically where it was before.

### "NERVOUS ECZEMAS."

Dr. L. D. Bulkley, in a reprint from *The Medical News*, describes many cases of eczema produced, kept up or excited by various nervous influences, as "nerve exhaustion," "nervous and mental shock," "reflex, of internal or peripheral origin," "structural and functional neuroses" and diseases of the brain and cord. Hence, in studying the general condition of an eczematous patient it is well to investigate the nervous system as well as the digestive and urinary. The fact is the "nervous disorder" may be the *cause of the cause* of eczema.

### COMEDO.

Dr. J. Abbott Cantrell, of Philadelphia, advises the use of electrolysis for the treatment of comedones (black-heads). The small papule with the comedo in the center, or the simple comedo without the papular condition, might be so treated, the idea doubtless being to cause resolution of the papule and possibly destruction of the offending sebaceous gland. The needle is attached to the negative, the sponge electrode to the positive pole. Five to twenty cells used, according to indications.

---

### PSORIASIS.

Dr. Bulkley (reprint from *Md. Med. Journal*, 1891) describes *psoriasis* as "a chronic affection of the skin, exhibiting dry, red, slightly elevated patches or spots, of varying size and shape, generally circular, covered with a greater or less quantity of dry, white, silvery scales, heaped together, the lesions tending to develop chiefly on the extensor surfaces." And he might have further mentioned the tendency to affect the elbows and knees, the almost characteristic deepening of the cutaneous lines in the patches, the pin point oozing of blood when the scales and their basement membrane were scratched off. The patches often run together making map-like figures. Arsenic in increasing doses, suspended when tolerance is reached, long continued; and the rubbing in of a chrysarobin salve, or one containing some one of the tarry preparations, offer the best hope of relief. Alkaline baths and scrubbing the patches with strong soap aid the treatment.

The ointment of the ammoniate of mercury gives good results when the scalp is affected. Useful, also, on the general surface, but if a large surface is covered salivation is likely to occur.

---

### LEECHES FOR ORCHITIS.

At the meeting of the Atlanta Society of Medicine on May 2, Dr. J. M. Glass reported the successful treatment of three cases of orchitis by the application of leeches to the most dependent part. All other treatment had failed.

Dr. H. P. Cooper had applied leeches along the swollen cord in a case of epididymitis. The pain was relieved, but the course of the disease was not influenced.

---

### REMOVAL OF AN EXTENSIVE HAIRY NÆVUS OF THE FACE BY ELECTROLYSIS.

Dr. Geo. Henry Fox, of New York, has an illustrated article upon this case in the May *Journal of Cutaneous and Genito-Urinary Diseases.*

The growth occupied the lower right eyelid, the cheek just below and the corresponding side of the bridge of the nose of a young man of twenty-two. It was dark, raised, warty-looking, mostly covered with a growth of coarse dark hair. The electrolytic needle was introduced through the most superficial parts of the growth and the current allowed to pass in the usual way. The hairs not thus destroyed were directly treated. Treatment extended over nearly four years. The result was practically perfect. A surgical operation would have been quicker, but the appearance left much less desirable.

---

### INJECTION OF IODOFORM OINTMENT IN SUPPURATING BUBOES.

In the May *Journal of Cutaneous and Genito-Urinary Diseases,* Dr. Wm. K. Otis, of New York, details this treatment as follows: The skin for eight or ten inches around was made thoroughly antiseptic. Abscess cavity opened with a narrow bistoury and contents thoroughly pressed out. Then washed out with 1 to 1000 bichloride. A warm 10 per cent. iodoform ointment then injected with an ordinary cone pointed glass syringe. Ointment at opening then solidified by cold compress of bichloride solution. Then the usual antiseptic dressings. Majority well in ten days.

### CHANCROIDAL BUBO OF THE AXILLA.

Infection of a frost-bitten finger from penis sore, followed by a bubo in the axilla. The pus from this produced a chancroid when inoculated upon the patient.—*Monatshefte für Prak. Derm. from An. de Derm. et de Syph.*

---

### FOR BOILS.

*Wiener Medicinische Wochenschrift*, 1893, No. 1, recommends the application of the following for boils.

℞. Chloral hydrate, ʒi.

Distilled water and glycerin, each ℥ij.

M. Saturate compresses with it and apply.

---

### VENEREAL SORES.

Don't cauterize a sore or excoriation on the penis until you find out its character, for the caustic action may mask the true nature of the lesion so as to leave your patient a lifetime of doubt.

For a herpes use a dry soothing powder, or the powder on absorbent cotton.

For a chancre, equal parts mercurial ointment and oxide of zinc ointment is a pretty good dressing, but you will not hasten the cure much.

Thorough cleansing of a chancroid and a good antiseptic dressing is useful. Caustics don't help matters much in any of these, and may do much harm in herpes.

---

### "TETTER."

When you tell your patient he or she has "tetter" you use a term which is fully as indefinite as "fever." The works on dermatology mention no disease of this name. You can't cure a "tetter" treated as a "tetter." There are two many tetters: *dandruff, psoriasis, ringworm, eczema, etc.*

